# A case of Castleman disease with hemophagocytic syndrome derived from HHV8 infection

**DOI:** 10.1186/s40001-021-00589-5

**Published:** 2021-10-03

**Authors:** Xiao Cui, Yongfeng Wu, Lin Jia, Jing Chang, Chuanyun Li, Caiping Guo, Tong Zhang, Yingmin Ma, Yulin Zhang

**Affiliations:** 1 Department of Infectious Diseases, Beijing You An Hospital, Capital Medical University, Beijing Institute of Hepatology, Beijing, 100069 China; 2 Pathology Diagnostic Center, Beijing You An Hospital, Capital Medical University, Beijing, 100069 China; 3 Department of Hepatobiliary Surgery and You An Liver Transplant Center, Beijing You An Hospital, Capital Medical University, Beijing, 100069 China; 4 Department of Respiratory and Infectious Diseases, Beijing You An Hospital, Capital Medical University, Beijing Institute of Hepatology, Beijing, 100069 China

**Keywords:** Human herpes virus-8, Multicentric Castleman disease, Hemophagocytic syndrome, Metagenomic sequencing technology

## Abstract

**Background:**

For a patient presenting with fever, multiple lymphadenopathy and splenomegaly, pathogen infection should be preferentially considered, followed by lymphoid malignancies. When traditional laboratory and pathological detection cannot find the pathogenic microorganism, metagenomic sequencing (MGS) which targets the person’s genome for exceptional genetic disorders may detect a rare pathogen.

**Case presentation:**

Here, we introduced the diagnostic clue of a case of multicentric Castleman disease (MCD) with hemophagocytic syndrome which was elicited from the detection of human herpesvirus-8 in the blood of a HIV-1 infected person by MGS technology during pathogen inspection. This case highlights the need to increase the awareness of MCD among clinicians and pathologists.

**Conclusions:**

MGS technology may play a pivotal role in providing diagnostic clues during pathogen inspection, especially when pathogens are not detectable by conventional methods.

## Background

Fever, multiple lymphadenopathy and splenomegaly often suggest pathogen infection or lymphoid malignancies. The ability to find the etiology of such symptoms timely is still lacking. Multicentric Castleman disease (MCD) is a rare disease that exhibit lymphadenopathy in > 1 lymph node station as well as a wide spectrum of clinical manifestations, including constitutional symptoms, fluid accumulation, cytopenia, and liver and kidney dysfunction [1]. It also represents a lymphoreticular complication of acquired immunodeficiency syndrome. Human herpes virus-8 (HHV8) was identified as an etiological driver of MCD [2]. Immune deficiency is the primary risk factor for MCD and investigators also noted an association between HIV and MCD [3]. Histopathologic examination of lymph node could help establish the diagnosis. However, if there is no sufficient clinical information provided for pathologist, no right diagnosis could be made. The nonspecific symptoms and conventional clinical practice usually could not make a timely and accurate diagnosis of MCD.

Here, we presented a case of HHV8-positive MCD with hemophagocytic syndrome (HPS) in HIV infection and introduced the diagnostic clue which was elicited from the detection of HHV8 in the plasma of a HIV-1 infected person by metagenomic sequencing (MGS) technology during pathogen detection.

## Case presentation

**Fig. 1:**
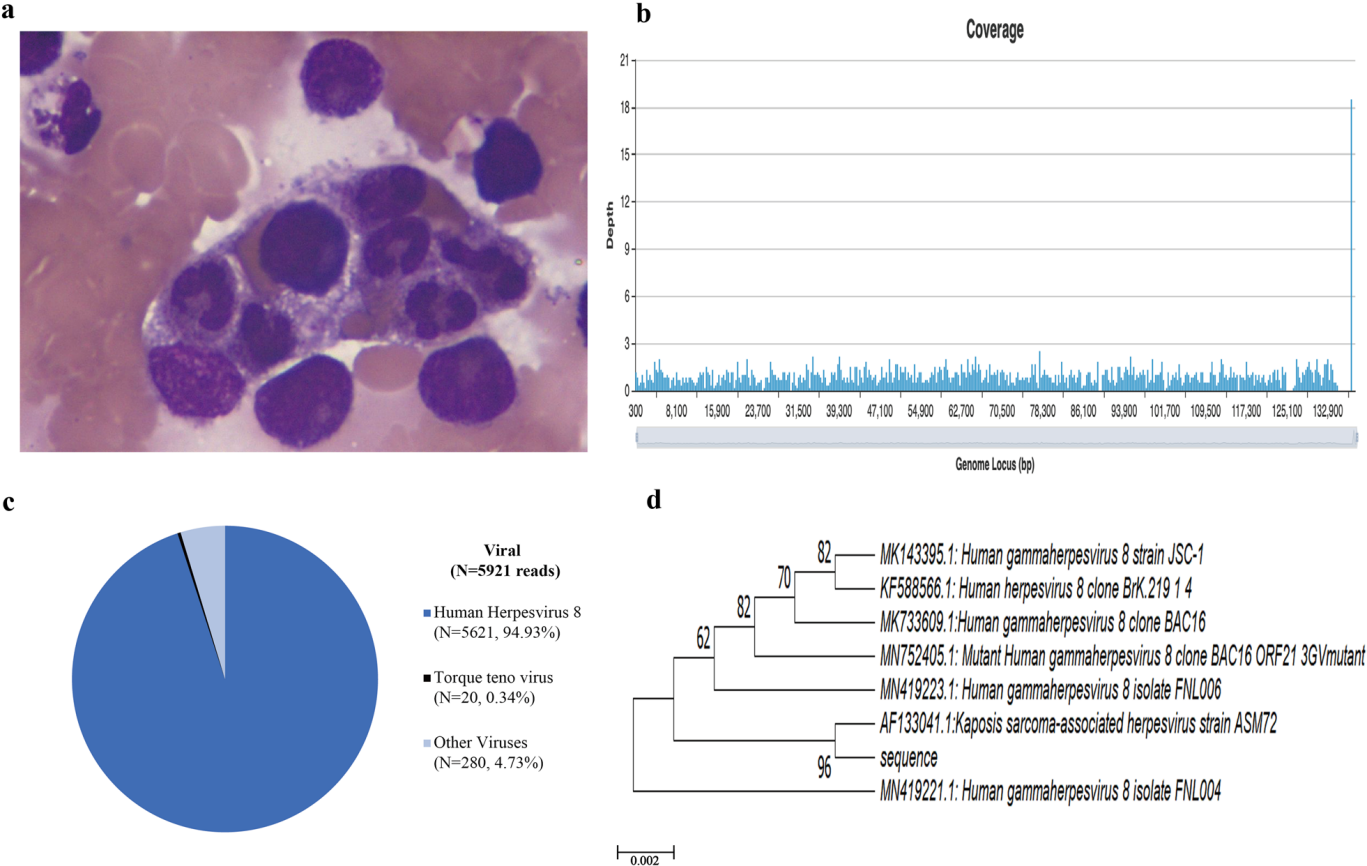
**a** Hemophagocytosis image in bone marrow. **b** Mapping of 5621 human herpesvirus-8 (HHV8) reads derived from the patient’s peripheral blood sample. **c** The distribution of viral sequences identified in the patient’s peripheral blood. **d** Phylogenetic analysis showed sequences from representative serovars, strains, and species of HHV8. The scale bar denoted the number of nucleotide substitutions per site

A 49-year-old man with confirmed HIV-1 infection 14 days before treated with lamivudine, tenofovir and efavirenz was admitted with a 3-month history of fatigue, weight loss and fever. He smokes occasionally but denies any alcohol or illicit drug use. He also reported unprotected sex. He has splenomegaly and generalized lymphadenopathy occurred in the neck, axilla, mediastinum and celiac on physical examination and computed tomography scan. A detailed clinical work-up for infection, immune and malignant diseases were performed. His CD4 count was 48 cells/μl and HIV viral load was 8596 copies/ml. Laboratory tests showed pancytopenia, elevated erythrocyte sedimentation rate and C-reactive protein, polyclonal hypergammaglobulinemia and hypoalbuminemia. Serological testing for cryptococcus, Epstein Barr virus, cytomegalovirus was unrevealing (Table 1). Furthermore, peripheral blood cultures incubated for 5 days were also negative. Hemophagocytosis could be seen on bone marrow smears (Fig. 1a), complied with fever, splenomegaly, three-line cytopenia, high level of serum ferritin and soluble CD25, supporting the diagnosis of HPS. A lymph node biopsy with the highest standardized uptake value (SUV = 6.0) provided by a positron emission tomography–computed tomography (PET–CT) scan revealed nonspecific lymphocyte proliferation. Pathological imaging of lymph node biopsy showed nonspecific lymphocyte proliferation and excluded malignancy.

**Fig. 2:**
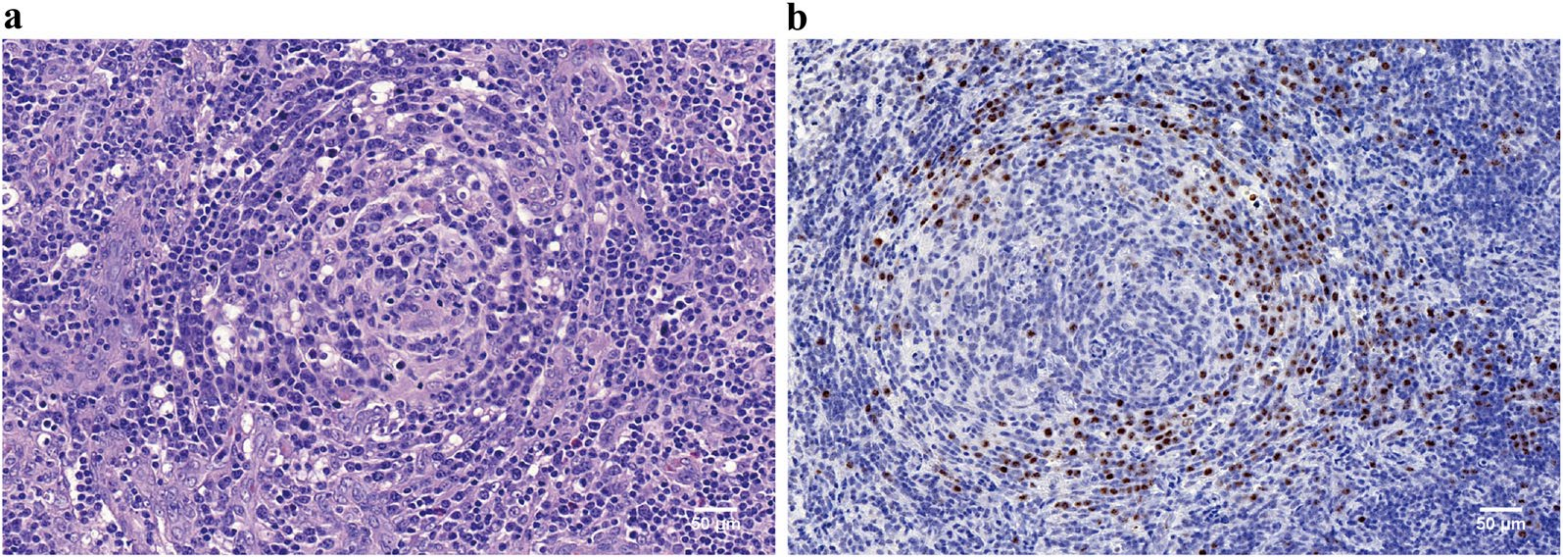
**a** Hematoxylin and eosin staining of lymph node showed plentiful plasma cell infiltration in perifollicular and mantle zone. The follicle had an atrophic germinal center. **b** Positive HHV8 expression in plasma cells localized in perifollicular and mantle zone by immunohistochemical staining (original magnification ×200)

To find the cause of HPS, a MGS assay of plasma was performed and the result revealed a HHV8 viremia of 5621 unique reads with coverage of identified viral genes 94.93% (Fig. 1b–d). HHV8-associated diseases were further considered. No Kaposi’s sarcoma evidence was found on the skin, oral and gastrointestinal mucosa by endoscopy. Then the evidence of HHV8 viral load test positive demonstration of in situ hybridization and histopathology evaluation on the lymph node tissue confirmed HHV8-associated multicentric Castleman disease (HHV8-MCD). It was characterized by the presence of sheets of plasma cell in the interfollicular zone. Prominent high endothelial venules could be observed in the interfollicular region. Moreover, the lymphoid follicles were dissolved, with atrophic germinal centers (Fig. 2a, b). Treatment with 6 cycles of rituximab, cyclophosphamide, doxorubicin, vincristine, and prednisolone and combined with ganciclovir for anti-HHV8 treatment in the context of HIV infection, improved the patient’s condition. Nevertheless, the Charlson Comorbidity Index was 8, which suggests poor prognosis.

**Table 1:** Laboratory test results on admission

Test item	Test value	Normal range
White blood cell counts (10^9^/L)	2.85	3.5–9.5
Neutrophil counts (10^9^/L)	1.26	2.0–7.5
Neutrophils percentage (%)	59.6	40–75
Lymphocyte percentage (%)	30.9	20–50
Hemoglobin (g/L)	82	130–175
Platelets (10^9^/L)	67	125–350
Blood urea (mmol/L)	6.68	2.9–8.2
Creatinine (μmol/L)	74	57–97
Alanine transarninase (U/L)	21	9–50
Glutamic–oxaloacetic transaminase (U/L)	27	15–40
Total bilirubin (μmol/L)	15.2	5–21
Direct bilirubin (μmol/L)	6.8	< 7
Albumin (g/L)	27.9	40–55
Triglycerides(mmol/L)	2.94	0.45–1.69
Lactate dehydrogenase (U/L)	242	120–250
CD4 cell counts (cells /μL)	48	600–800
Erythrocyte sedimentation rate (mm/hr)	70	0–15
High-sensitivity C-reactive protein (mg/L)	167.3	0–3
Procalcitonin (ng/mL)	2.98	< 0.1
Plasma (1,3) beta-d-glucan (pg/mL)	28.3	< 60
Serum galactomannan antigen	Negative	Negative
Cryptococcus antigen	Negative	Negative
Anti-EBV-EA immunoglobulin M antibody	Negative	Negative
Anti-EBV-VCA immunoglobulin M antibody	Negative	Negative
Anti-CMV immunoglobulin M antibody	Negative	Negative
Anti-Mycoplasma immunoglobulin M antibody	Negative	Negative
Anti-Chlamydia immunoglobulin M antibody	Negative	Negative
EBV DNA (copies/mL)	< 500	< 500
CMV DNA (copies/mL)	< 500	< 500
HIV RNA loads(copies/mL)	8596	< 500
Soluble CD25(IU/mL)	952.4	13.1–43.7
Serum Ferritin(μg/L)	2902	15–200

EBV: Epstein Barr virus, CMV: cytomegalovirus, EA: early antigen, VCA: viral capsid antigen, HIV: Human Immunodeficiency Virus

## Discussion and conclusions

HHV8 infection is found to correlate with Kaposi's sarcoma (KS) and hematologic diseases, including primary effusion lymphoma and MCD [4]. Here, we have documented a case of HHV8-MCD which was diagnosed based on the detection of HHV8 in the plasma of a HIV-1 infected person by MGS technology and histopathologic findings. It was difficult to diagnosis without HHV8 stains because its histological features can be similar to other diseases such as HIV lymphadenitis.

For a case of fever unknown origin, pathogenic microorganism inspection commonly does not include HHV8 detection. But specific HHV8 detection is suggested for a HIV-1 infected patient with fever and lymphatic hyperplasia, because immunodeficiency increases the risk of HHV8 infection. MGS assay may provide a valuable diagnostic support for HHV8 infection [5]. Further, for a HIV/AIDS patient with HHV8 co-infection, KS and Castleman disease (CD) should be considered [6]. HPS is a reactive disorder of the reticuloendothelial system, and occasionally be found in HIV/AIDS patients with malignancies, severe opportunistic infections or autoimmune diseases [7]. CD with HPS is extremely rare and it is difficult to differentiate HPS and CD due to their similar presentations [8]. Thus, HHV8 finding may provide a diagnostic clue for CD with HPS.

To our knowledge, previously documented case of HHV8-associated MCD was mainly diagnosed by the histologic confirmation of lymph node [9, 10]. However, the diagnostic clues and process remain unclear. Here, we presented the diagnostic procedure of the infectious disease, which may provide reference for the diagnosis of unusual pathogens.

In conclusion, physicians should be expanding the knowledge for diseases presenting with fever and lymphadenopathy and be aware about the possibility of MCD in patients with immunodeficiency. The diagnosis might be difficult, but the etiology should be repeatedly sought. MGS may provide a diagnostic clue for infection-associated diseases.

## Abbreviations

MGS: Metagenomic sequencing
MCD: Multicentric Castleman disease
HHV8: Human herpes virus-8
HPS: Hemophagocytic syndrome
PET–CT: Positron emission tomography–computed tomography
HHV8-MCD: HHV8-associated multicentric Castleman disease
KS: Kaposi's sarcoma
CD: Castleman disease

## Declarations

### Availability of data and materials

Data sharing is not applicable to this article as no datasets were generated or analyzed during the current study.
